# A hands-free carotid Doppler can identify spontaneous circulation without interrupting cardiopulmonary resuscitation: an animal study

**DOI:** 10.1186/s40635-024-00704-w

**Published:** 2024-12-27

**Authors:** Bjørn Ove Faldaas, Benjamin Stage Storm, Knut Tore Lappegård, Ole-Jakob How, Bent Aksel Nilsen, Gabriel Kiss, Eirik Skogvoll, Erik Waage Nielsen, Hans Torp, Charlotte Björk Ingul

**Affiliations:** 1 Faculty of Nursing and Health Sciences, Nord University, Bodø, Norway; 2 Department of Circulation and Medical Imaging, Norwegian University of Science and Technology (NTNU), Trondheim, Norway; 3 Department of Clinical Medicine, Faculty of Health Sciences, UiT the Arctic University of Norway, Tromsø, Norway; 4 Department of Surgery, Nordland Hospital Trust, Bodø, Norway; 5 Department of Pain Medicine and Research, Oslo University Hospital, University of Oslo, Oslo, Norway; 6 Department of Medicine, Nordland Hospital Trust, Bodø, Norway; 7 Faculty of Biosciences and Aquaculture, Nord University, Bodø, Norway; 8 Department of Medical Biology, Faculty of Health Sciences, UiT the Arctic University of Norway, Tromsø, Norway; 9 Department of Computer Science (IDI), Faculty of Information Technology and Electrical Engineering, Norwegian University of Science and Technology (NTNU), Trondheim, Norway; 10 Clinic of Anesthesia and Intensive Care Medicine, St Olav University Hospital, Trondheim, Norway

**Keywords:** Cardiac arrest, Chest compression, Cardiopulmonary resuscitation, Feedback device, Return of spontaneous circulation, Cardiac arrest monitor, Ultrasound, Carotid artery blood flow, Doppler

## Abstract

**Background:**

Identifying spontaneous circulation during cardiopulmonary resuscitation (CPR) is challenging. Current methods, which involve intermittent and time-consuming pulse checks, necessitate pauses in chest compressions. This issue is problematic in both in-hospital cardiac arrest and out-of-hospital cardiac arrest situations, where resources for identifying circulation during CPR may be limited. The fraction of chest compression plays a pivotal role in improving survival rates. To address this challenge, we evaluated a newly developed hands-free, continuous carotid Doppler system (RescueDoppler), designed to identify spontaneous circulation during chest compressions. In our study, we utilized a porcine model of cardiac arrest to investigate sequences of ventricular fibrillation, followed by defibrillation, and monitoring for the return of spontaneous circulation during chest compressions with the carotid Doppler system. We explored both manual compressions at 100 and 50 compressions per minute and mechanical compressions. To estimate the detection rate (i.e., sensitivity), we employed a logistic mixed model with animal identity as random effect.

**Results:**

Offline analysis of Doppler color M-mode and spectral display successfully identified spontaneous circulation during chest compressions in all compression models. Spontaneous circulation was detected in 51 of 59 sequences, yielding an expected sensitivity of 98% with a 95% confidence interval of 59% to 99%.

**Conclusion:**

The RescueDoppler, a continuous hands-free carotid Doppler system, demonstrates an expected sensitivity of 98% for identifying spontaneous circulation during both manual and mechanical chest compressions. Clinical studies are needed to further validate these findings.

## Background

The European Resuscitation Council (ERC) emphasizes the importance of high-quality chest compressions with minimal interruptions [1], pausing only for rhythm analysis and checking for return of spontaneous circulation (ROSC) when there is a combination of clinical and physiological signs [1]. Studies have shown that a higher chest compression fraction can improve the chances of achieving ROSC [2, 3]. Identifying circulation during circulatory collapse, during resuscitation, and after ROSC can be extremely challenging. Conditions such as pseudo-pulseless electrical activity (pPEA) and other situations with extremely low blood pressure are especially difficult to manage. Commonly used methods, such as manual palpation of the pulse, have limitations related to reliability and are time-consuming [4, 5].

Ultrasound is widely used during cardiopulmonary resuscitation (CPR) today [1, 6]. Recent research has focused on using Doppler and hands-free systems to identify circulation and monitor hemodynamics during resuscitation. Nevertheless, the interpretation of chest compression data is constrained by the occurrence of movement artifacts [7–14].

To address the challenges associated with the lack of hands-free and continuous ultrasound systems, we have developed a hands-free Doppler system, RescueDoppler, designed to detect carotid blood velocities during resuscitation continuously [11, 13]. Studies on porcine models have demonstrated that the RescueDoppler is capable not only of identifying pulsatile blood flow even at blood pressures below 60 mmHg [11] but also of identifying blood velocities generated by chest compressions. Further, during chest compressions, RescueDoppler has the potential to identify the hand position yielding the highest time average velocity (TAV) [13].

This study investigates whether the RescueDoppler system can identify spontaneous circulation during various chest compression models in a porcine cardiac arrest model. Manual compressions at 100 or 50 per minute and mechanical compressions were used.

## Methods

### Instrumentation

At the certified pathogen-free farm, the porcine (*Sus scrofa domesticus*) underwent sedation utilizing an intramuscular administration of 1000 mg ketamine (Ketalar, Pfizer AS, Norway), 1 mg atropine (G.L. Pharma GmbH, Lannach, Austria), and 5 mg midazolam (B. Braun Melsungen AG, Germany), prior to their transfer to the laboratory. Anesthesia was initiated with intravenous midazolam and morphine (G.L. Pharma GmbH, Lannach, Austria) and sustained with intravenous infusions of morphine, midazolam, and thiopental (Pentocur Abcur AB, Sweden). The sedation and anesthesia protocols were executed following the detailed protocol described earlier [11]. Mechanical ventilation of the pigs was performed using a GE Engstøm Carestation ventilator (GE Healthcare), with parameters set at 21% FiO_2_, a tidal volume of 10–15 mL/kg, a respiratory rate of 13 to 16 breaths per minute, and a positive end-expiratory pressure set to zero. A TruWave pressure monitoring transducer kit (Edwards Lifesciences Corporation, Irvine, CA) was interfaced with the arterial cannula. To mitigate the risk of catheter occlusion, 500 mL of pressurized Ringer’s acetate (Baxter AS, Norway) with the addition of 1250 IU of heparin (LEO Pharma AS, Norway) was administered with a flow rate of 3 ± 1 mL/h. The procedures for managing and instrumenting the pigs have been detailed in previous paper [11].

### Monitoring

End-tidal CO_2_ (ETCO_2_) levels were monitored, and minute ventilation was modulated to uphold a pH level of 7.4. The animals’ core temperature was monitored to maintain within the range of 38.5–39.0℃. Continuous monitoring included a 5-lead electrocardiogram (ECG), invasive arterial and central venous pressures, and peripheral oxygen saturation (SpO_2_).

### RescueDoppler system

The RescueDoppler is a pulsed wave Doppler system, integrating a tailor-made carotid Doppler probe, a dedicated scanner (Manus EIM-A, Aurotech Ultrasound AS), a laptop equipped with MATLAB (R2022b) for real-time visualization, and a user interface.

This system utilizes dual probes, each positioned at fixed angles of + 30 and −30 degrees, as detailed in earlier publication [11]. The non-focused transducer is 6 × 30 mm with a central frequency of 4 MHz. The system uses 32 depth ranges spanning between 8 and 45 mm [11]. In the post-processing phase, angle correction is calculated from the velocity curve derived from each probe. Additionally, the system displays color M-mode and Doppler spectral for the chosen depth and calculates peak systolic velocity and TAV from each cardiac cycle or velocity curve. Doppler data are synchronized with the ECG, invasive blood pressure, ETCO_2_, and central venous pressure, following established protocols [11].

The probe was positioned and fixated with self-adherent tape over the carotid artery, and Doppler signals were confirmed. The complete experimental setup and data collection methods are described in previous studies [11]. Velocity spikes and baseline noise in the Doppler spectra from tissue motion during compressions were removed using digital wall and clutter filters in MATLAB. Doppler velocities below 30 cm/s were adjusted to 0 cm/s to compensate for tissue movement during chest compressions.

### Ventricular fibrillation model

Ventricular fibrillation (VF) was induced with an implantable cardioverter-defibrillator (ICD) (St. Jude Medial Ellipse DR Model CD2377-36C, Merlin Patient Care System, Abbott). The ICD lead was positioned in the ventricular apex via the external jugular vein, guided by echocardiography (GE Vivid S7 Pro, 3S probe). The ICD unit was then implanted subcutaneously in the upper right chest. VF was initiated through the “DC Fibber” induction technique using St. Jude Medical´s system, and a 7.5-V direct current shock was administered for 2 s [15]. Subsequent defibrillation with 30 J was performed with the ICD to restore ROSC.

### Chest compression models

**Fig. 1:**
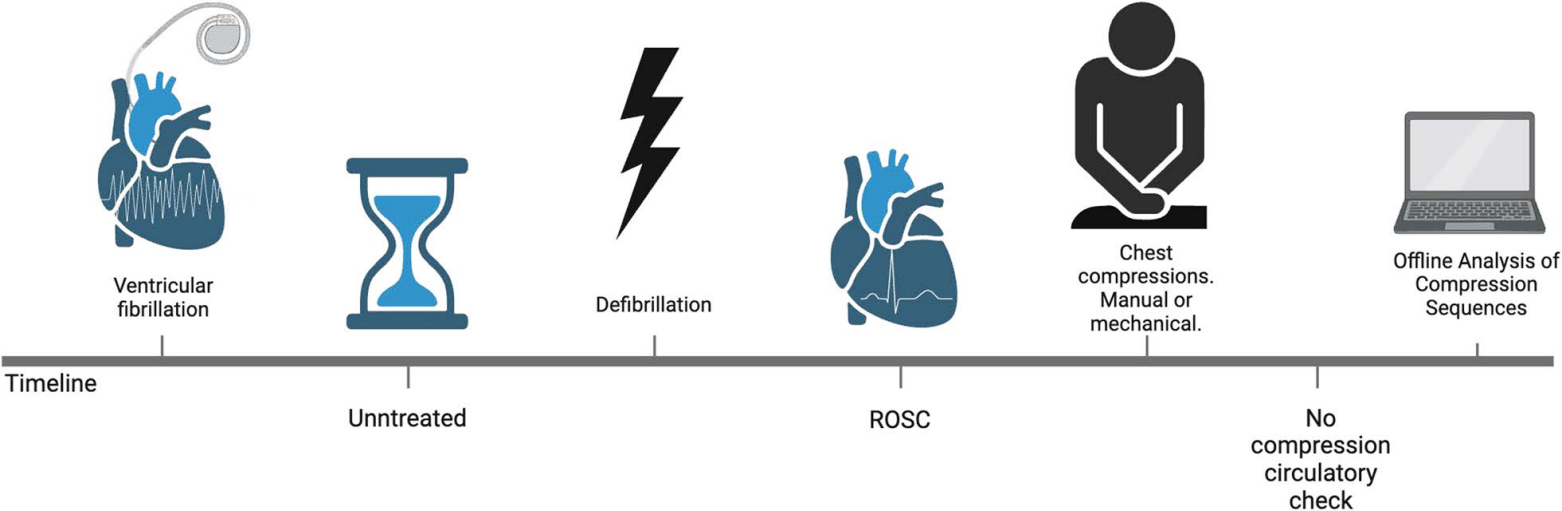
This timeline illustrates the chest compression sequence protocol. First, ventricular fibrillation was induced by ICD. Next, the animals were left untreated for 30 to 60 s. Following this, defibrillation was administered. After defibrillation, the animals received either manual or mechanical chest compressions for 15 to 60 s. Subsequently, a circulation check was performed without compressions to confirm ROSC using ECG and invasive blood pressure. Finally, the compression sequences were analyzed offline. Created with BioRender.com

After the induction of VF, the pigs were left untreated for 30 to 60 s before receiving defibrillation, which was followed by either manual or mechanical chest compressions, with a duration of 15 to 60 s (Fig. 1). Subsequently, a circulation check was performed without compressions to confirm ROSC using ECG and invasive blood pressure measurements. ROSC was identified by the presence of an organized heart rhythm on the ECG and a pulsatile arterial blood pressure greater than 60 mmHg. Ventilations were paused during chest compressions. Prior to and after each chest compression sequence, FiO2 was increased to 100% to avoid hypoxia. There was a 5-min interval between chest compression sequences.

### Manual compressions with 100 and 50 compressions per minute

Chest compressions with a frequency of 100 compressions per minute (CPM) were performed by one trained rescuer (BF). This leaves an observation period for detecting spontaneous circulation between each compression of about 0.6 s, assuming a duty cycle of 50%. With manual compressions at 50 CPM, the observation period increases to 1.2 s. A metronome (“The metronome” by Soundbrenner App Store 2020) was used to maintain compression frequency.

### Mechanical compressions

Mechanical compressions were performed with the LUCAS-3 (Lund University Cardiopulmonary Assist System) chest compression system (Stryker, 2825 Airview Boulevard Kalamazoo, MI, 49,002 USA), which provides 102 ± 2 CPM. The compression piston was placed centrally on the lower part of the sternum, perpendicular to the animal’s thorax. The area around the suction cup was marked with a marker to allow for adjustments if it moved from its original position.

### Myocardial infarction model

Myocardial infarction (MI) was induced by left coronary microembolization using 50 µm polystyrene microspheres (Chromosphere, Thermo ScientificTM) in a 0.9% sodium chloride and 0.01% Tween 20 solution [16]. The model aimed to decrease systolic pressure to 60 mmHg or below, as previously described in detail [11].

### Doppler analysis

**Fig. 2:**
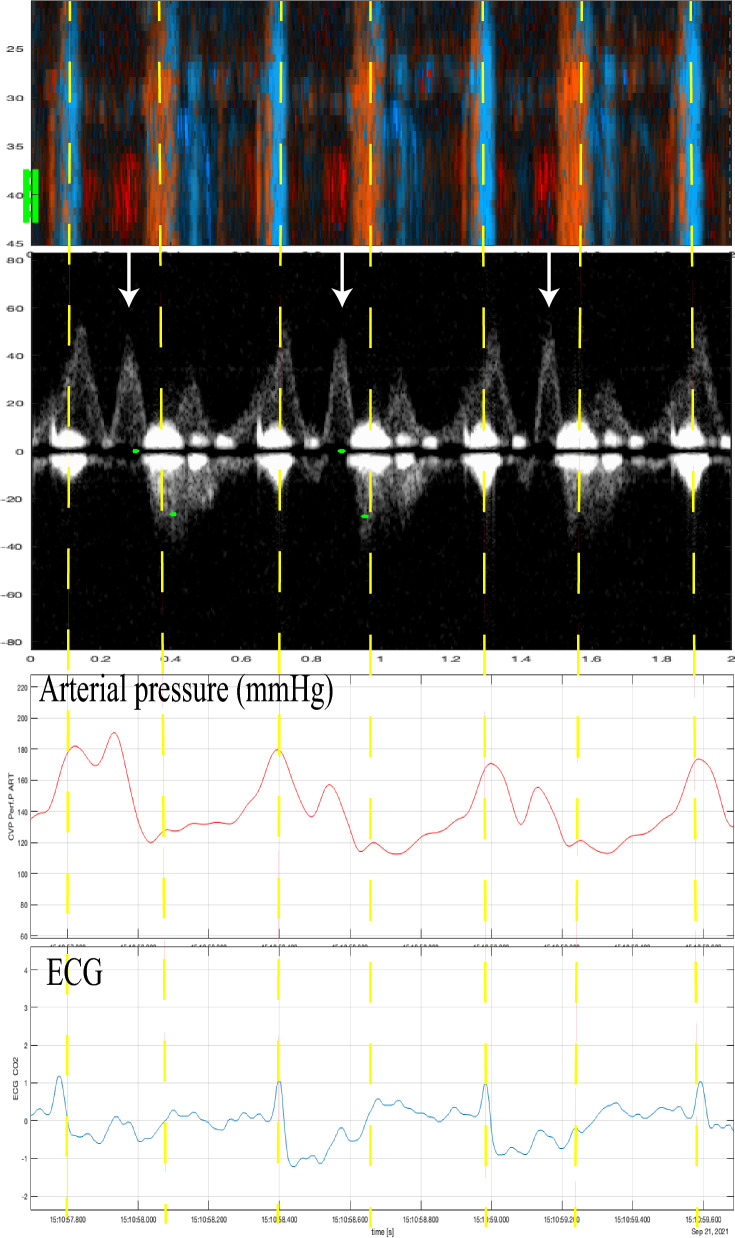
Example of ROSC detection analysis during mechanical chest compressions. This sequence spans two seconds and includes four panels: the first panel shows a Color M-mode, the second panel displays the corresponding Doppler spectrum from about 40 mm, the third panel presents arterial pressure (mmHg), and the fourth panel shows the ECG. The vertical dashed line indicates compressions identified as continuous velocity curves in M-mode and motion artifacts in the baseline of Doppler spectrums. Arrows in the spectrum highlight signs of ROSC

**Fig. 3:**
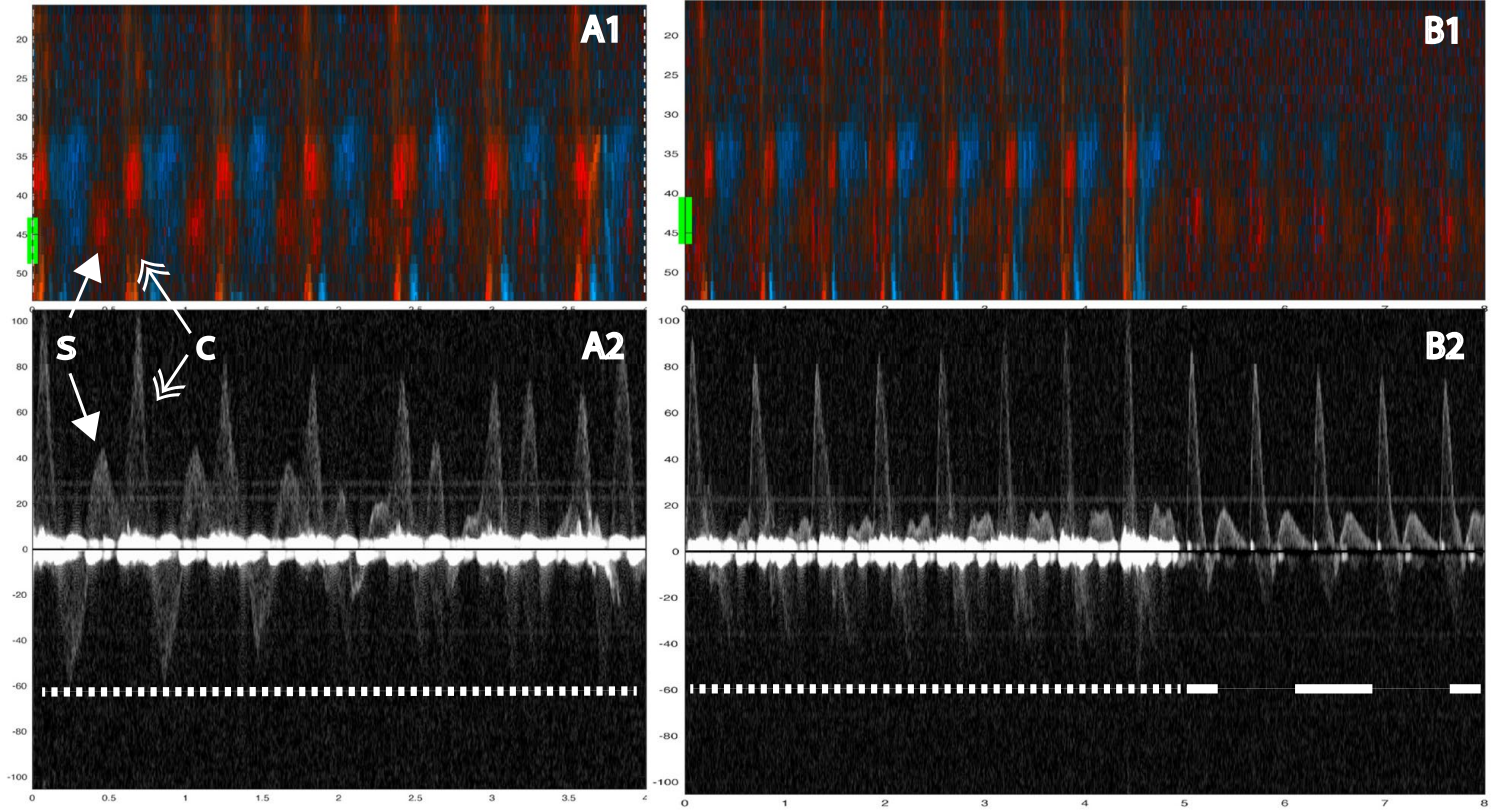
Two examples showing evidence of spontaneous circulation. In A1 (color M-mode) during manual compressions at 100/min, one sees spontaneous circulation appearing as red signals at a depth of about 40 mm (yellow line at the margin). The corresponding Doppler spectrum (A2) from the chosen depth of 40 mm shows spontaneous velocity signals interspersed between (and out of phase with) the compression-generated spectra. In B1 (color M-mode) during manual compressions at 100/ min, first one sees compression (tissue) artifacts across the entire depth obscuring the spontaneous signals, which mainly become evident during the circulatory check. In B2 the spontaneous velocity signals are immediately visible at the circulatory check. Below both panels, a narrow-dashed line indicates ongoing chest compressions, and a wide-dashed line indicates a circulatory check **B**. The two double arrows: the single-tip arrows indicate spontaneous circulation (S), and the double-tip arrows indicate chest compression (**C**). Velocities are in cm/s

**Fig. 4:**
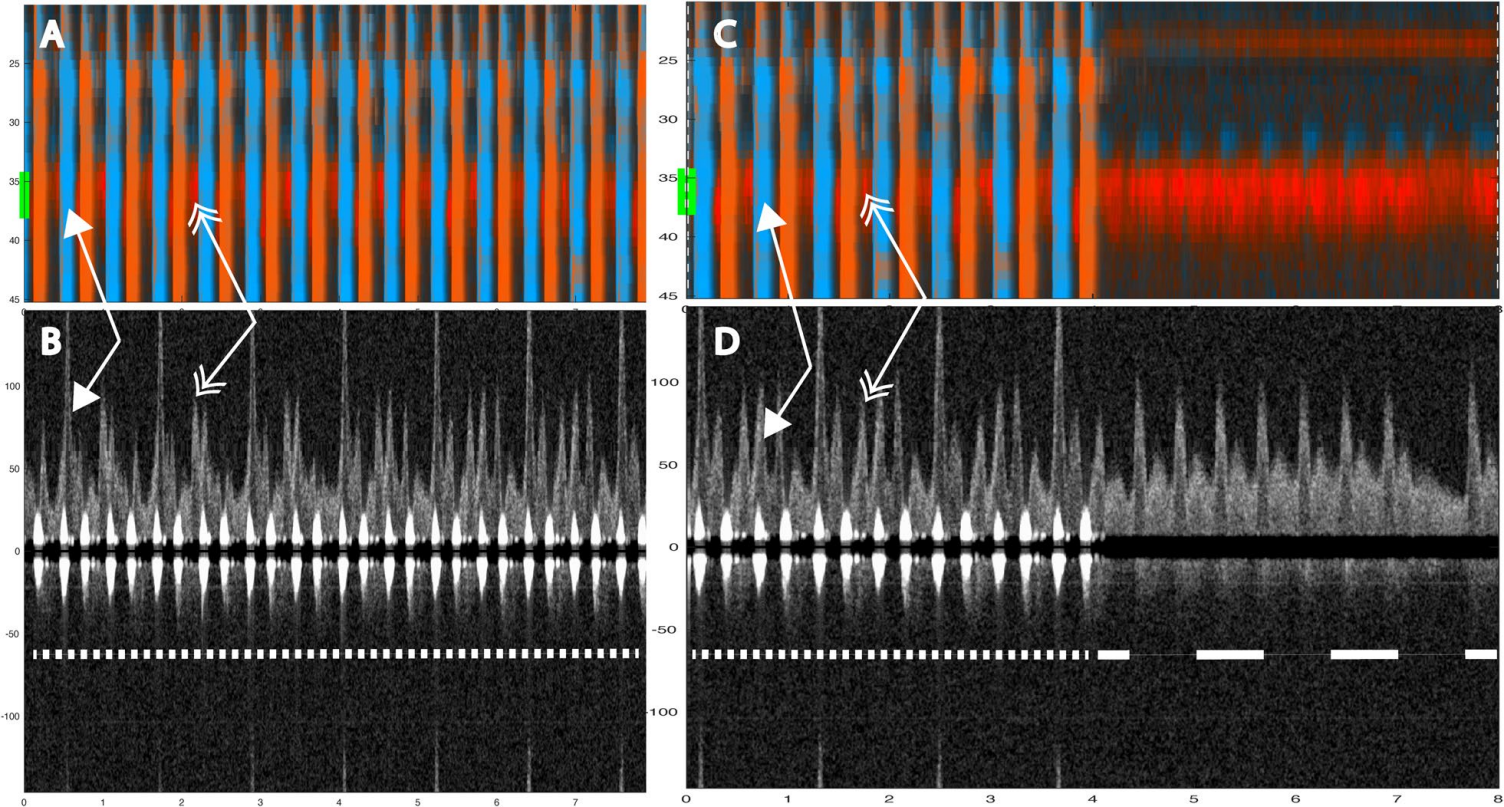
Two 8-s examples showing evidence of spontaneous circulation. In A1 (color M-mode) during manual compressions at 50/ min, one sees spontaneous circulation appearing as red signals at a depth of about 37 mm (yellow line at the A1 and B1 margin). The corresponding Doppler spectrum (A2) from the chosen depth of 37 mm shows spontaneous velocity signals interspersed between the compression-generated spectra. In B1 (color M-mode) during manual compressions, first one sees compression (tissue) artifacts. Spontaneous circulation become evident during the circulatory check. In B2 the spontaneous velocity signals are immediately visible at the circulatory check. Below both panels, a narrow-dashed line indicates ongoing chest compressions, and a wide-dashed line indicates a circulatory check (panel B2). The two double arrows: the single-tip arrows indicate spontaneous circulation (S), and the double-tip arrows indicate chest compression (**C**). Velocities are in cm/s

The compression sequences were analyzed offline. First, compression artifacts were identified in color M-mode by consistent vertical motion velocities occurring simultaneously in the same direction across multiple depth ranges. Furthermore, we specifically looked for distinct and significant motion artifacts in the Doppler spectrum, particularly around the baseline and at the start of each chest compression. Then, spontaneous circulation between compressions was identified by a color M-mode-signal towards the probe (red) with corresponding motion velocities at the carotid depth (Figs. 2, 3). Chest compressions generate a high forward peak systolic blood flow velocity. The presence of spontaneous circulation during compressions was confirmed by detecting velocity curves during circulatory checks when no compressions were provided, as seen in Figs. 3, 4.

### Statistics

Continuous data are presented as mean, standard deviation (SD), 95% confidence interval, min, and max as appropriate. Discrete data are presented as counts and percentages. Sequences with spontaneous circulation after defibrillation were assessed using the RescueDoppler method and assigned 1 if ROSC was detected and 0 otherwise. To estimate the RescueDoppler method’s detection sensitivity, observations were entered into a logistic mixed effects model with animal identity as a random factor and the Huber–White variance estimator that adjusts for clustering of sequences within animals. The sample size was determined a priori by the available resources. We planned for lab availability over 9 days with one animal per day and a realistic data acquisition of up to ten sequences in each animal. This would yield a maximum sample size of 90. While we believed sensitivity to be high, the expected precision (i.e., the width of the 95% confidence interval) would also depend on the actual value and the intra-animal correlation. These were hard to specify in advance and not pursued further. Continuous and discrete data were exported to Excel Version 16.85 (Microsoft for Mac) before conducting the statistical analyses in Stata version 18 (College Station, Texas, USA).

## Results

**Fig. 5:**
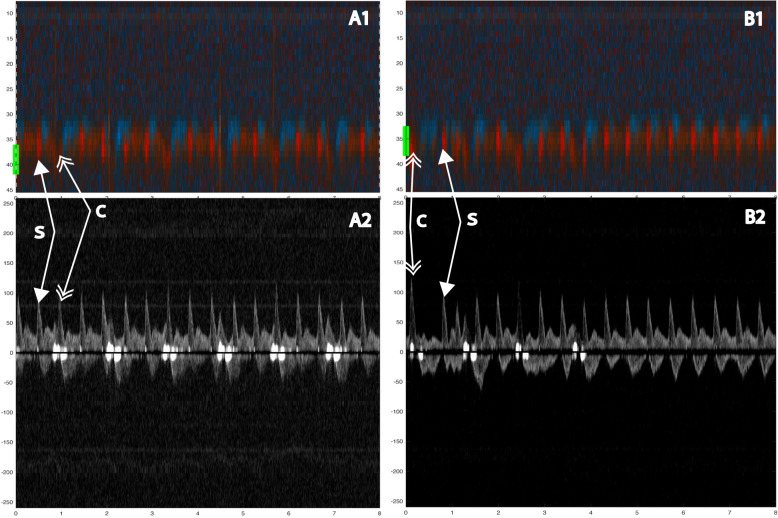
Color m-mode showing spontaneous circulation during mechanical compression. Two eight-second examples. Color M-mode (**A**, **C**) with the corresponding Doppler specter from the chosen depth below. Velocities are given in cm/s. In **B** and **D**, a narrow-dashed line indicates chest compressions and a wide-dashed line indicates a circulatory check (**B**, **D**). The two arrows: the single-tip arrow indicates spontaneous circulation, and the double-tip arrow indicates chest compression. M-mode (**A**) from 8-s mechanical 100 compressions with corresponding Doppler specter (**B**). After 4 s, the transition from compressions to circulatory check (**C**, **D**)

**Fig. 6:**
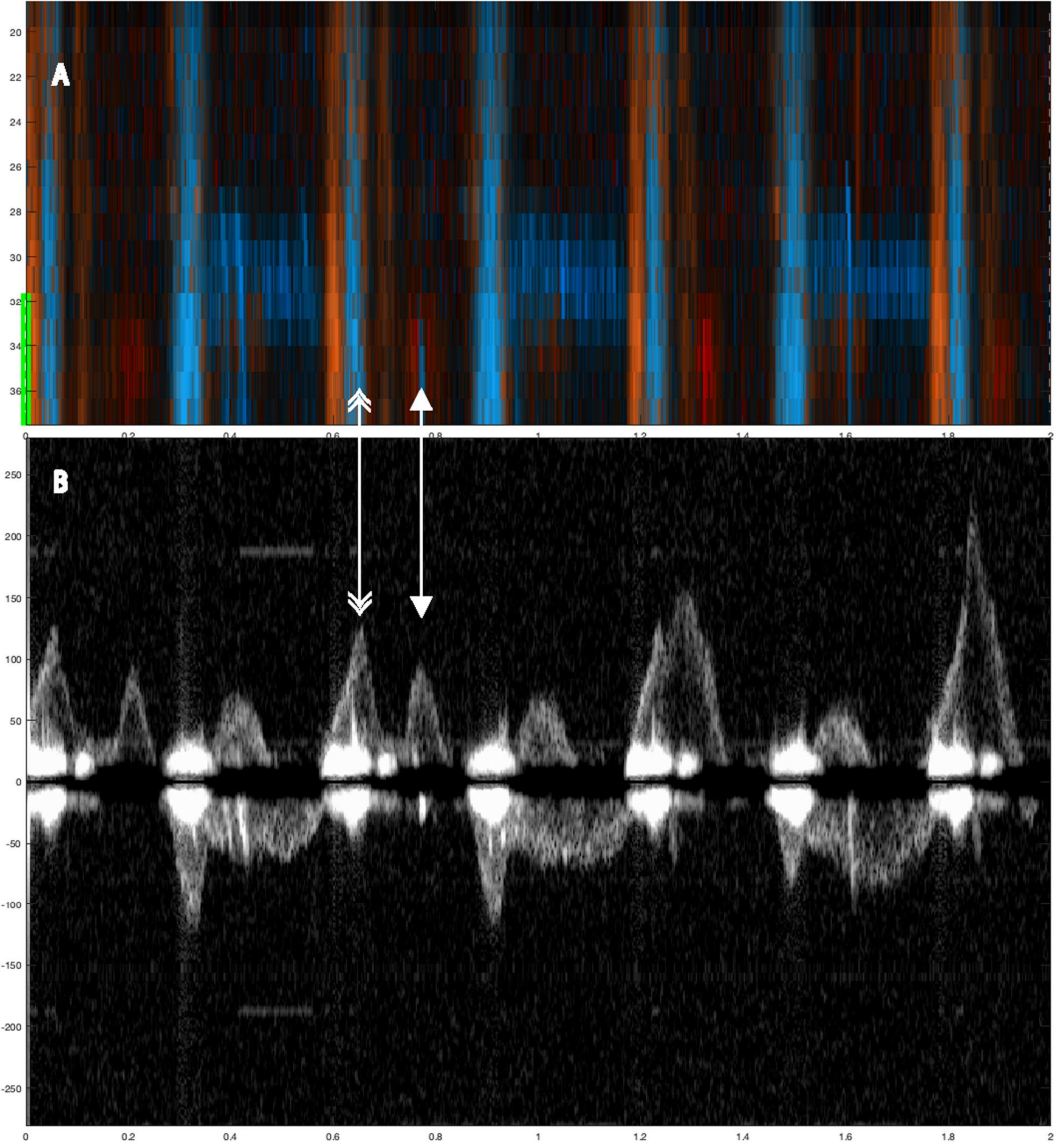
Two-second color M-mode and Doppler Spectrum showing spontaneous circulation during mechanical compression. Color M-mode (**A**) with the corresponding Doppler specter (**B**) from the chosen depth below. The figure includes two types of arrows: single-tip arrows indicate spontaneous circulation (S), while double-tip arrows denote chest compressions (**C**) Velocities are given in cm/s

**Fig. 7:**
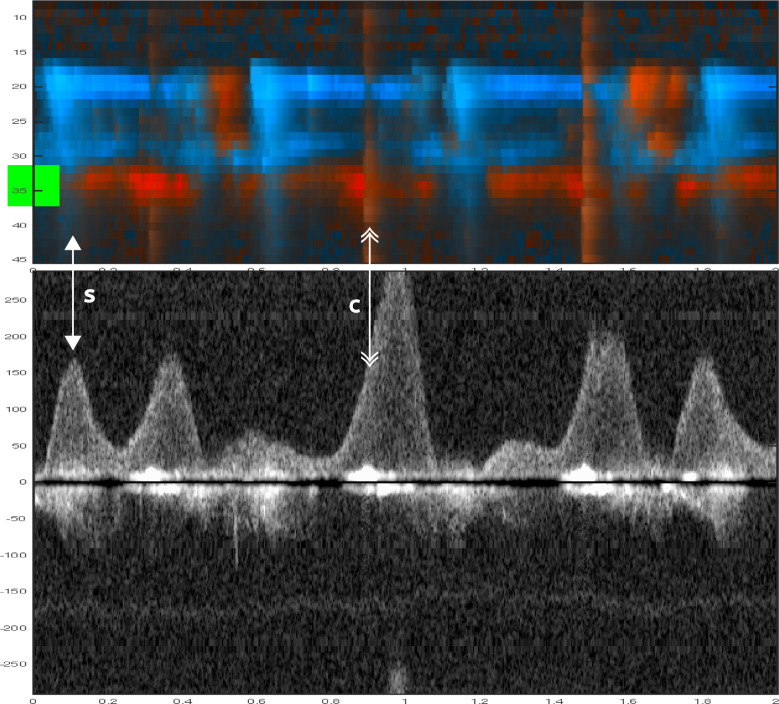
The upper panel (M-mode) and lower panel (Doppler spectra) demonstrate spontaneous circulation during mechanical compressions, captured over 2 s in an animal with myocardial infarction. The single-tip arrow indicate spontaneous circulation (S), and the double-tip arrow indicate chest compression (**C**). Velocities are given in cm/s

Nine animals and 59 chest compression sequences were included in the study (Table 2). Animal characteristics and baseline data are presented in Table 1. Carotid blood flow velocity and arterial pressure during chest compression and no compression are shown in Table 2. Mechanical compressions generated higher peak velocity, TAV, and peak pressure compared to manual compressions, but MAP was the same (Table 2). We identified chest compressions consisting of continuous vertical red/blue lines in the color M-mode spectrum (Figs. 2, 3, 4, 5, 6 and 7) as well as motion noise in the Doppler curve around the baseline at the start of the compression of the Doppler curve (Fig. 6). Although the compression rate was 100 CPM for both mechanical and manual compressions, manual compressions resulted in less tissue movement.

**Table 1:** Baseline characteristics of the nine animals

	Weight (kg)	Carotid (mm)	Depth (mm)	Thorax (cm)	Psyst (mmHg)	MAP (mmHg)	Pdiast (mmHg)	PSV (cm/s)	TAV (cm/s)
Mean	29.8	4.4	22.9	65.9	115.7	86.3	65.2	113.3	43.7
SD	1.8	0.5	6.7	1.9	12.7	8.5	8.1	16.2	16.9
Min	27.4	3.7	19.0	63.0	95.2	73.0	54.7	89.4	28.2
Max	32.0	5.0	40.0	68.5	136.0	94.9	77.8	145.4	80.8

Carotid: carotid diameter, depth: carotid depth, Psyst: peak systolic pressure, MAP: mean arterial pressure, Pdiast: peak diastolic pressure deviation, PSV: peak systolic velocity, TAV: time averaged velocity, SD: standard deviation, Min: minimum, Max: maximum

**Table 2:** Hemodynamics during chest compressions and with no compressions

		Mean	SD	95% confidence interval	
Mechanical compressions *n* = 30^1^	Vpeak	152	71	126	178
	TAV	38	28	27	48
	Ppeak	172	32	160	184
	MAP	71	16	65	77
Manual compressions 100 CPM *n* = 10	Vpeak	94	37	67	121
	TAV	19	24	1	36
	Ppeak	146	37	119	172
	MAP	70	21	55	85
Manual compressions 50 CPM *n* = 16	Vpeak	103	34	84	121
	TAV	20	15	12	27
	Ppeak	136	21	125	147
	MAP	65	20	54	75
No compressions *n* = 56^1^	Vpeak	105	56	90	120
	TAV	45	27	38	53
	Ppeak	121	18	116	125
	MAP	86	18	81	91

SD: standard deviation, Vpeak: peak velocity (cm/s), TAV: time averaged velocity (cm/s), Ppeak: peak pressure (mmHg), MAP: mean arterial pressure (mmHg), CPM: compressions per minute

^1^The total number of sequences was 59, but no data was obtained in 3 sequences

**Fig. 8:**
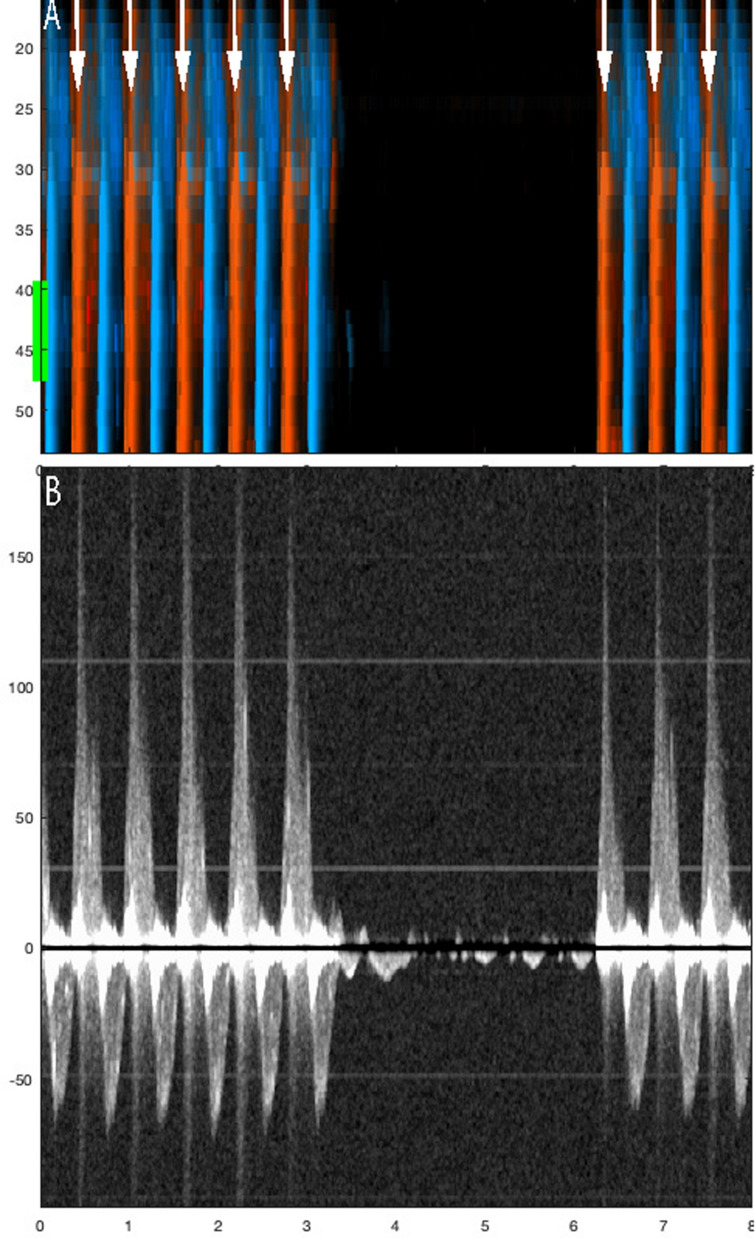
Circulation check with no spontaneous circulation during mechanical compressions. This figure illustrates the sequence of mechanical compressions performed during ventricular fibrillation when there is no spontaneous circulation. **A** (Color M-mode) and its corresponding Doppler spectrum (**B**) from a selected depth are shown. Arrows indicate chest compressions. A pause is made for circulation check after three seconds, and compressions are resumed at the six-second mark. The example is derived from a sequence that was not included in this study

During chest compression, we were able to identify the start of compression, peak positive flow, TAV, and peak negative flow (Fig. 6). Spontaneous circulation between chest compressions was characterized by the appearance of a velocity curve and sometimes diastolic flow at the end of the velocity curve (Fig. 4). Figure 8 illustrates an example of mechanical compressions without spontaneous circulation. The absence of blood pressure was confirmed during the circulatory check.

The carotid Doppler identified spontaneous circulation in 51 of 59 sequences from nine animals, with an average of 7 (range: 2 to 13) sequences per animal, yielding a sensitivity of 86%. However, failure occurred in two animals where ROSC was identified in 1 of 6 and 2 of 5 sequences, respectively. The expected sensitivity reported by the logistic mixed effects model with animal identity as a random factor is therefore higher at 98% (95% CI: 59% to 99%) because it is weighted towards the majority of animals. As every animal achieved ROSC, the specificity of RescueDoppler could not be determined. During 100 CPM, spontaneous circulation was identified in 10 out of 10 sequences (100%) (Fig. 3) and in 15 out of 16 sequences (94%) during manual compressions at a rate of 50 CPM (Fig. 3). During mechanical compressions, spontaneous circulation was identified in 26 out of 33 (79%) sequences (Figs. 5, 6) In the MI model using only mechanical compressions, spontaneous circulation was identified in six of six sequences (100%) from two animals (Fig. 7). ROSC was identified after all 59 sequences.

## Discussion

This study demonstrates the ability to identify spontaneous circulation during chest compressions using a hands-free carotid Doppler system. Spontaneous circulation was identified by differentiating between compression-generated and spontaneous velocities using a combination of Doppler spectra and color M-mode.

Continuous non-invasive hemodynamic Doppler monitoring of carotid velocity has the potential to identify spontaneous circulation during chest compressions, minimize unnecessary interruptions, and enhance efficiency during circulatory checks.

Identification of spontaneous circulation was achievable for all chest compression models. The main challenge was distinguishing between spontaneous blood velocities and compression-generated velocities and understanding how these influence each other. Compressions generated a distinctive pattern in the color M-mode and motion artifacts around the baselined or at the start of each chest compression in the Doppler spectrum (Fig. 3). By analyzing various depths in color M-mode (Fig. 3), we could differentiate compression movement from spontaneous circulation.

It was possible to identify spontaneous circulation when compression-generated and spontaneous velocities were sufficiently separated in time. This irregularity in velocity patterns could suggest cardiac contractility, indicating the presence of underlying spontaneous circulation. If compressions occur simultaneously with spontaneous circulation, one will overshadow the other in the flow curve. Furthermore, if the compressions or spontaneous circulation occur closely together, the first one will affect the next (Fig. 5).[17].

One animal study found that chest compressions, performed in the early post-ROSC period, potentially could be detrimental to hemodynamic function [18]. If compressions and spontaneous circulation are synchronous, signs of spontaneous circulation will not be observed. If spontaneous circulation is present, it can be detected if compressions are paused for circulatory check, if the compression rate is changed, or if compressions and spontaneous circulation become asynchronous over time.

Manual compressions generated less distinct motion in color M-mode and less tissue noise in the Doppler spectrum compared to mechanical compressions (Fig. 3). Mechanical compressions by the LUCAS-3 suction cup can contribute to active decompression. This was observed to generate more tissue movement, making it more challenging to interpret Doppler signals. Identifying spontaneous circulation during mechanical compressions was challenging due to tissue movements generating noise in the Doppler spectrum. Spontaneous circulation was observed in the spectrum, but active decompression generated motion velocities in M-mode, complicating confirmation. Manual compressions resulted in less tissue movement than mechanical compressions, which made it easier to identify spontaneous circulation.

One factor that affects the identification of spontaneous circulation during chest compressions is the observation time between compressions. We, therefore, reduced the compression rate to 50 CPM to increase the observation time between compressions to 1.2 s (Fig. 4). Reducing the compression rate to increase observation time may potentially enhance the chances of detecting spontaneous circulation without halting compressions, but this was not observed in this study.

This animal study provides valuable insights for the further development of the RescueDoppler system. Failures to identify blood velocities occurred in two out of nine animals, likely due to multiple factors, including but not limited to, the attachment of the probe was not optimized, variations in the animals’ neck anatomy, the probe losing contact with the skin during compression movements, and insufficient amounts of ultrasound gel. We acknowledge the challenges of using RescueDoppler in clinical settings, such as probe placement difficulties and noise interference from ongoing CPR. Additionally, the failure to detect velocities due to insufficient contact between the probe and skin in this study highlights the need for further refinement. These issues must be addressed through advancements in both probe and software development, specifically improving the probe attachment. Conducting clinical pilots and studies is an essential future step. By focusing on these areas, we aim to enhance the reliability and effectiveness of the RescueDoppler in clinical environments.

Future clinical studies should examine whether the moment chosen to stop compressions to check for spontaneous circulation and the duration spent identifying spontaneous circulation can be reduced by identifying spontaneous circulation during compressions.

### Summary

This animal study used a hands-free carotid Doppler system to identify spontaneous circulation during chest compressions. Both manual compressions at 100 and 50 CPM and mechanical compressions were used. In total, 59 sequences from nine animals were analyzed for signs of spontaneous circulation. Analysis was done with color M-mode and Doppler spectra. Spontaneous circulation was confirmed if velocity curves were identified between or connected to velocity curves from chest compressions.

### Limitations

This study has several limitations. The animals studied were young and healthy. Although there are similarities between human and porcine hemodynamics, it is essential to recognize differences, such as the shape of the chest and the anatomical difference of the neck. It is imperative that the probe is securely fastened to the neck to prevent loss of skin contact or positional changes due to movements performed by compressions, which was the reason why some data could not be analyzed and were not included. Another limitation of the study is the non-blinded design, which could introduce positive bias in favor of RescueDoppler when assessing its accuracy in detecting ROSC.

Analyzing Doppler data has inherent limitations, as manual adjustment of gain and sample volume can affect results. The RescueDoppler system functions the same whether it is used to measure animals or humans.

## Conclusions

Identifying spontaneous circulation during various forms of chest compression is possible using the RescueDoppler, a hands-free, continuous carotid Doppler system. The sensitivity for detecting spontaneous circulation during chest compressions was 98%, with a 95% confidence interval of 59% to 99%. Clinical studies are needed to investigate the clinical utility of RescueDoppler and its implications during CPR.

## Data Availability

The datasets used and analyzed during the current study are available from the corresponding author upon reasonable request.

## Abbreviations

VF: Ventricular fibrillation
ROSC: Return of spontaneous circulation
CPM: Compressions per minute
CI: Confidence interval
PEA: Pulseless electrical activity
pPEA: Pseudo-pulseless electrical activity
ERC: European Resuscitation Council
TAV: Time average velocity
mL: Milliliter
mL/hr: Milliliter per hour
ETCO_2_: End-tidal CO_2_
ECG: Electrocardiogram
ETCO_2_: End-tidal carbon dioxide
ICD: Implantable cardioverter-defibrillator
SD: Standard deviation
